# Use of Tachycardia in Patients With Submassive Pulmonary Emboli to Risk Stratify for Early Initiation of Thrombolytic Therapy: A Case Series Comparing Early Versus Late Thrombolytic Initiation

**DOI:** 10.1177/2324709617744232

**Published:** 2017-12-11

**Authors:** Jordana Cheta, Ashleigh Long, Paul Marik

**Affiliations:** 1 Eastern Virginia Medical School, Norfolk, VA, USA

**Keywords:** pulmonary embolism, tachycardia and PE, right ventricular strain and PE, submassive pulmonary embolism, thrombolytics in pulmonary embolism, intermediate-risk pulmonary embolism

## Abstract

Pulmonary embolism (PE) represents a prevalent cause of morbidity and mortality in the United States, with approximately 600 000 cases diagnosed annually. The mortality rate for untreated PE is as high as 30%. Right ventricular (RV) dysfunction is a sign of possible adverse outcomes with right-sided heart failure being the usual cause of death from PE. There is a spectrum of clinical presentations associated with PE diagnoses, from incidental and asymptomatic to rapid hemodynamic collapse. Despite successes in identifying patients with “high-risk” PEs for aggressive thrombolytic interventions and “low-risk” PEs for outpatient anticoagulation, a significant lack of consensus exists regarding intervention modalities for PEs identified as “intermediate risk” or “submassive,” defined as normotensive (systolic blood pressure ≥90 mm Hg) with acute RV dysfunction and myocardial injury. In this case series, we review the management and outcomes of 2 patients with submassive PEs and sustained tachycardia in the setting of normal blood pressures, and we address the need to recognize tachycardia as an ominous RV compensatory sign, indicative of impending hemodynamic collapse, that should lead to aggressive therapy with vascular intervention.

## Introduction

While anticoagulation versus thrombolytic use is relatively straightforward when treating patients with low-risk PE (defined as normotensive with normal biomarker levels and no RV dysfunction on imaging) and high-risk PE (shock and/or low blood pressure, defined as a systolic blood pressure <90 mm Hg or a pressure drop of 40 mm Hg for >15 minutes, if not caused by new-onset arrhythmia, hypovolemia, or sepsis), it is not so straightforward for patients in the intermediate-risk PE groups.^1-5^

RV dysfunction will require the presence of at least one of the following:

- RV dilation (apical 4-chamber RV diameter divided by left ventricular [LV] diameter >0.9) or RV systolic dysfunction on echocardiography
- RV dilation (4-chamber RV diameter divided by LV diameter >0.9) on computed tomography (CT)
- Elevation of brain natriatic peptide (BNP; >90 pg/mL) in absence of heart or renal failure
- Elevation of N-terminal pro-BNP (>500 pg/mL) in absence of heart or renal failure
- Electrocardiographic changes (new complete or incomplete right bundle-branch block, anteroseptal ST elevation or depression, or anteroseptal T-wave inversion)

Myocardial necrosis is defined as either of the following:

- Elevation of troponin I (>0.4 ng/mL)
- Elevation of troponin T (>0.1 ng/mL)^5^

Current management guidelines state systemic administration of antithrombolytic therapy be reserved for patients with “high-risk” or “massive” PE causing hemodynamic instability.^6-8^ Conversely, patients with “low-risk” PEs are typically ideal candidates for outpatient management.^7,9^ Adding to the complexity of PE risk-stratification is the “intermediate-risk” or “submassive” presentations. This category, in particular, has raised a number of debates regarding optimal management, including identification of those most likely to decompensate, even in the setting of systemic anticoagulation.^9,10^

Multiple scoring systems have been developed to risk stratify patients having increased risk of PE-related outcomes. The Pulmonary Embolism Severity Index is a clinical model that identifies low-risk PE patients for outpatient treatments. The Wells and revised Geneva scores can be used to objectively identify the risk of a suspected PE, both incorporating heart rate. When the pulmonary arterial pressures increase as a result of an obstruction by an embolus, the RV afterload increases, in turn resulting in RV dilation, and ultimately decreased cardiac output and possible death.^11^ Furthermore, in the setting of acute PE, the presence of RV dysfunction without evidence of hemodynamic compromise has been associated with a 2- to 10-fold higher risk of early mortality, compared with normotensive patients with acute PE but without RV dysfunction.^6^

Markers of RV dysfunction, such as echocardiogram, spiral CT, BNP, and myocardial injury (positive cardiac troponin testing), have low predictive values for PE complications when used alone.^12^ Bova et al^13^ developed a statistically significant prognostic grading system to predict PE-related complications in intermediate-risk PE populations, which combined clinical presentation, RV dysfunction, and myocardial injury. Predictors of complications included systolic blood pressure 90 to 100 mm Hg, heart rate ≥110 beats per minute (BPM), and elevated cardiac troponin levels.^13^ This study found that the combination of the above-mentioned parameters predicted a 7-fold increase in the risk of an adverse 30-day PE-related outcome.^13^ Findings on echocardiogram suggest RV dysfunction include RV dilatation, RV hypokinesis with apical motion sparing, flattening or paradoxical movement of the interventricular septum toward the LV in diastole, pulmonary hypertension, and tricuspid regurgitation.^14^ Computed tomography pulmonary angiography (CTPA) is currently the gold standard in diagnosing PEs. A RV/LV diameter ratio is also easily determined by CTPA, and the 30-day mortality rate is 15.6% in patients with CTPA and RV/LV >0.9 compared with 7.7% in patients without RV enlargement.^15^

Clinical suspicion for the presence of RV failure should be for every patient admitted with acute PE, regardless of initial hemodynamic stability.^6,7,9^

Herein, we review 2 similar cases that involved presentations of intermediate-risk pulmonary emboli, where both patients displayed persistent tachycardia in the setting of normal blood pressures. We review the outcomes of both cases, and we discuss why persistent tachycardia is a compensatory measure indicative of impending hemodynamic collapse in the setting of PE.

## Case 1

**Figure 1.**
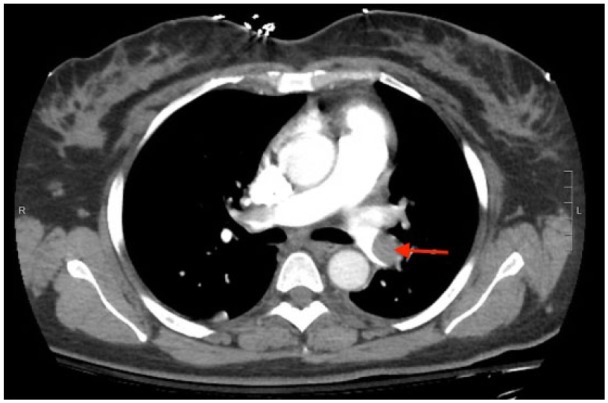
CTPA demonstrating extensive pulmonary emboli throughout the distal left pulmonary artery (red arrow) extending into the upper and lower lobe branches.

**Figure 2.**
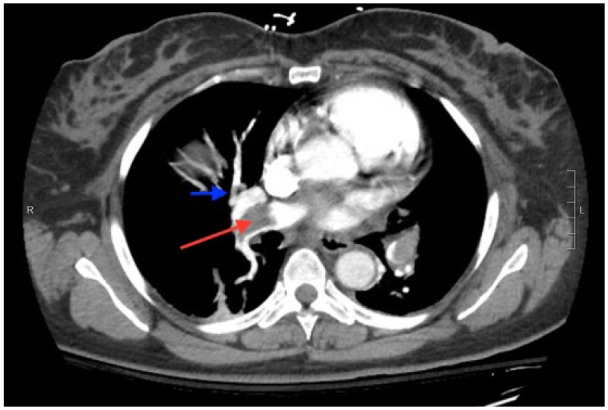
CTPA demonstrating extension of right-sided emboli, in the right lower, mid, and upper lobe pulmonary artery (red arrow) extending into segmental (blue arrow) and subsegmental branches.

We report a case of a 53-year-old African American woman with multiple sclerosis admitted for the placement of a L5-S1 intrathecal baclofen pump for spastic paralysis. The procedure and immediate postoperative course were uncomplicated. Her past medical history was significant for anxiety, essential hypertension, and type 2 diabetes mellitus without additional complications. As discharge preparations were being made, the patient’s heart rate was noted to be acutely elevated. Her vital signs were as follows: temperature 98.8°F, blood pressure 111/80 mm Hg, respiratory rate 32 breaths per minute, oxygen saturation (SpO_2_) 90% on room air, and a persistent heart rate of 138 BPM. A 2-liter bolus of lactated Ringer’s solution was administered as well as 0.5 mg of oral lorazepam with no consequent change in heart rate. The patient denied any pain or distress on bedside examination; physical examination was remarkable for increased tone and spasticity of lower extremities with 5/5 strength and normal light touch. Laboratory values were significant for a pro-BNP of 2898 pg/mL, and an arterial blood gas result of pH 7.4, partial pressure of carbon dioxide (pCO_2_) 33.4, and partial pressure of oxygen (PaO_2_) 53.0. Complete blood count, thyroid function panel, and basic metabolic panel collected that morning were unremarkable. Electrocardiogram was significant for sinus tachycardia, and Doppler studies of the lower extremities were negative for evidence of deep vein thrombus bilaterally. Subsequently performed echocardiogram revealed normal LV systolic function, ejection fraction of 55%, flattened septum in diastole, and mildly dilated RV size with severely reduced systolic function, moderate tricuspid regurgitation, and a mild increase in pulmonary artery pressures to 42 mm Hg. CTPA was positive for extensive pulmonary emboli throughout the distal left pulmonary artery extending into the upper and lower lobe branches (Figure 1). Additional right-sided emboli, in the right lower, mid, and upper lobe pulmonary artery, extending into segmental and subsegmental branches, were additionally noted (Figure 2). The patient was diagnosed with bilateral submassive pulmonary emboli, and anticoagulation with a heparin drip was started and vascular surgery was consulted. She was subsequently taken for emergent catheter-directed thrombolysis secondary to noted indications of impending hemodynamic compromise, which included a heart rate greater than 110 BPM, elevated pro-BNP, echocardiogram findings of right heart strain, and extensive CTPA findings. Following thrombolysis, the patient’s recovery was uneventful, with resolution of tachycardia and subsequent discharged to rehabilitation on apixaban for maintenance anticoagulation. Her repeat CTPA 10 months later demonstrated total resolution of pulmonary emboli.

## Case 2

**Figure 3.**
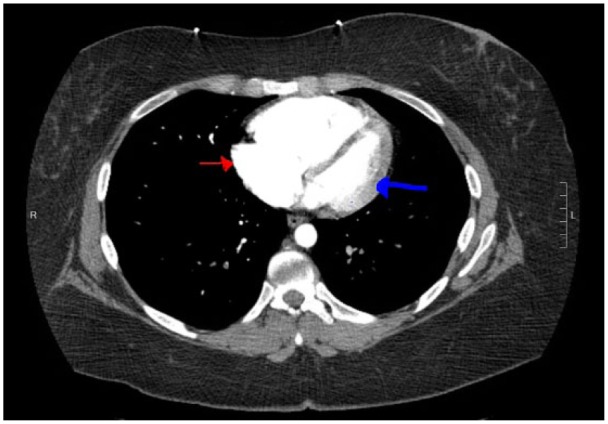
CTPA showing a dilated RV, with a LV/RV ratio greater than 2.0.

**Figure 4.**
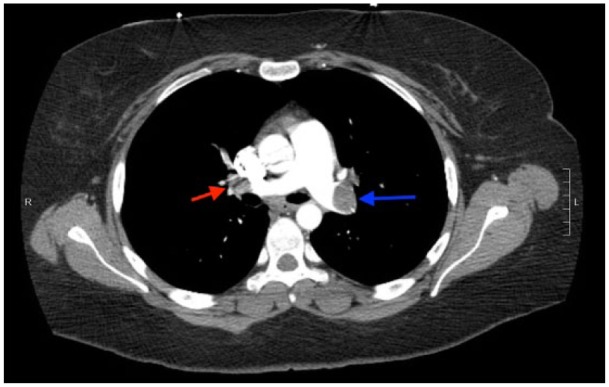
CTPA with extensive embolic filling defects throughout the pulmonary vasculature. Significant embolic filling defects are noted in the right (red arrow) and left (blue) main pulmonary artery.

A 31-year-old woman presented to the emergency room with shortness of breath and pleuritic chest pain that began the evening prior. Her past medical history was significant for hypothyroidism, obesity (status post gastric sleeve surgery in the year prior), and included previous incidence of a left lower extremity deep vein thrombus following a knee injury, managed with 6 months of anticoagulation, since completed. Her medication list at time of presentation consisted of levothyroxine and medroxyprogesterone acetate. Vital signs on admission were noted as follows: blood pressure 120/86 mm Hg, heart rate 136 BPM, respiratory rate 25 BPM, and SpO_2_ of 94%. Physical examination was unremarkable with symmetric, nontender lower extremities. Laboratory investigations were significant for elevations in pro-BNP (866 pg/mL), troponin T (0.10 mg/mL), and creatinine kinase-MB index (66%). Complete blood count and basic metabolic panel were unremarkable. Electrocardiogram was significant for sinus tachycardia. Chest X-ray was unremarkable. Lower extremity Doppler scans were ordered and were noted to be positive for acute deep venous thrombosis in the right popliteal, posterior tibial, and peroneal veins. While the echocardiogram was pending, the cardiac phase of the CTPA allowed for the indirect assessment of RV function. On the CTPA the RV was dilated; the septum bowed with a LV/RV ratio greater than 2.0. The RV and LV diameters were manually measured as the maximum distance from the interventricular septum to the endocardial border, perpendicular to the long axis of each ventricle (Figure 3). These findings were diagnostic of RV dysfunction. The CTPA also revealed significant clot burden with extensive embolic filling defects throughout the pulmonary vasculature. All lobes were involved, and there were significant embolic defects in the right and left main pulmonary artery (Figure 4).

The patient was immediately put on a heparin drip (80 units/kg bolus then infusion starting at 18 units/kg/h) and admitted to the general medicine floor. Within 4 hours from the time of admission, the patient became acutely agitated, diaphoretic, and tachypneic, with vital signs as follows: 140/86 mm Hg, heart rate of 160 BPM, and SpO_2_ of 85%. She was ordered for 2 mg oral lorazepam, transferred to the intensive care unit, and placed on high-flow nasal cannula with improvement to SpO_2_ to 96%. Within 1 hour and 20 minutes of transfer, patient cardiac arrested.

Cardiopulmonary resuscitation was performed for 1 hour, with 100 mg of intravenous tissue plasminogen activator administered for her PE. The code was ultimately unsuccessful, and the patient subsequently passed.

## Discussion

When the pulmonary vasculature is impaired with large acute pulmonary emboli, RV dysfunction commonly develops as a consequence of increased pulmonary vascular resistance. The increase in pulmonary artery pressure leads to an increase in RV afterload, RV dilation, and progressive RV failure. This results in reduced cardiac output and ultimately is the cause of death in most PEs. As a compensatory measure, consistently elevated heart rates in patients with PE act to maintain cardiac output, with normotensive blood pressures and adequate systemic perfusion. This, however, cannot be sustained, as we report here with our patient case series.

The clinical evaluation of these patients is of paramount importance to establish early diagnosis. Data from the ICOPER registry have also shown that patients with RV dysfunction on echocardiogram have an increased risk of death.^10^ In the PIOPED II study, conducted between 2001 and 2003, multidetector CTPA had a sensitivity of 83% and a specificity of 96%, and when used with CT venography of the lower limbs, this increased to 90% and 95%, respectively.^16^ The PROJECT (Prognostic Value of CT Scan in Haemodynamically Stable Patients with Acute Symptomatic PE) study also assessed RV dysfunction by CT and used predefined cutoff point to determine abnormal versus normal RV function.^17^

The PEITHO trial, the largest randomized, double-blinded trial published to date, investigated the role of thrombolysis in intermediate-risk PE patients, which was defined as the presence of RV dysfunction on echocardiography or CT together with myocardial injury.^10^ Patients were randomized to receive tenecteplase plus heparin versus placebo plus heparin. The use of anticoagulation alone in normotensive patients with PE was supported, but the use of thrombolysis in this intermediate-risk cohort could not be supported uniformly, given an elevated risk-benefit ratio involving catastrophic events, such as major intracranial bleeding.^10^ Separate meta-analyses reiterate this elevated risk of bleeding with thrombolytic management of intermediate-risk PEs but suggest that risk of bleeding events may be reduced in patients less than 65 years of age.^17^

Several recent studies have suggested that treatment with thrombolysis plus anticoagulation had significantly improved clinical outcomes. The MOPPETT trial, a randomized, placebo-controlled trial, compared the effects on reduction of pulmonary artery pressure with treatment with heparin plus tissue plasminogen activator versus heparin alone on patients with intermediate-risk PE.^18,19^ In this study, clot burden on CTPA, and not echocardiography, was used to define intermediate PE. Only 20% of the patients showed RV dilation on initial echocardiogram.^18^ The thrombolytic group demonstrated significantly lower pulmonary artery systolic pressures, but the clinical implications of these findings are unclear.

We discuss here the management of 2 recent cases of pulmonary emboli, both stratified into an “intermediate-risk” category secondary to evidence of right heart strain with normal blood pressures, with the only clinical indicator of hemodynamic compromise, from massive clot burden in the pulmonary vasculature, being persistent tachycardia. The patient in the first case received emergent catheter-directed thrombolytic therapy and had an uneventful course. The patient in the second case received heparin following admission, with brief documentation of possible consideration for nonurgent vascular intervention with catheter-directed thrombolytic therapy. Her cardiovascular status significantly worsened over the course of 4 hours following admission, and despite late administration of systemic thrombolytic therapy given as a resuscitative effort, the patient ultimately expired.

Despite being normotensive, tachycardia is an ominous sign of possible impending cardiovascular collapse. It suggests severe reduction in RV function, with a very low RV stroke volume with the blood pressure being maintained by the elevated heart rate, with heart rate increase being proportional to the degree of RV dysfunction. Rapid restoration of pulmonary artery flow can improve pulmonary perfusion, reduce pulmonary pressures, and prevent cardiac failure. Mechanical techniques and catheter-directed thrombolysis will result in quicker clot dissolution than systemic thrombolysis.^20^

## Conclusion

With recognition of tachycardia as an early indicator of hemodynamic compromise and an unsustainable compensatory measure of diminished RV cardiac reserve, earlier consideration for catheter-directed thrombolytic therapy should be initiated in patients with tachycardia and submassive PE before onset of hemodynamic instability requiring salvage measures with systemic thrombolytic therapy.
